# New Normal for Medical Practice Post COVID-19?

**DOI:** 10.1089/pop.2020.29003.nas

**Published:** 2020-10-16

**Authors:** David B. Nash

**Affiliations:** Jefferson College of Population Health, Philadelphia, Pennsylvania, USA.

As I write, the United States has been held captive for the better part of a year. Across the nation, COVID-19 has spawned fear, anxiety, disruption, and tragedy. It also has forced professionals in every sector to confront new realities and reassess the ways in which they attend to “business as usual.”

The traditional “art” of medicine relies heavily on face-to-face contact - close visual inspection, touch/palpation, picking up on a wide range of sensory and behavioral diagnostic clues. However, the critical need for social distancing to reduce the spread of COVID-19 has accelerated an inevitable shift from in-person to virtual communication. One positive consequence of this is that it forces us to take a hard look at the way we practice medicine.

Already an accepted mode of health care delivery in the United States, there is convincing evidence that telehealth is efficient, effective, and beneficial for specific uses and patient populations.^1^ This supplement focuses on one such population.

A substantial population of women with symptoms of vaginitis make up to 10 million outpatient visits per year^2^ with associated costs approaching $1.3 billion.^3^ The current standard of care calls for a visual examination of external and internal structures as well as vaginal secretions, a “whiff test” to detect distinctive odors associated with specific infections, and on-site pH and wet mount diagnostic tests. A bimanual exam is also recommended.

There are several common causes of vaginitis, and differential diagnosis is challenging even under “normal” conditions. The literature suggests that a large percentage of patients do not receive an optimal diagnostic evaluation. The expected shift from in-person to virtual outpatient visits likely will further complicate the diagnostic dilemma.

To me, the implications are clear: it is time to reconsider how best to approach care for this patient population under “the new normal” conditions. There is an obvious synergy between telehealth visits and state-of-the-art molecular technology that can be leveraged to improve the efficiency and accuracy of care we provide to women with vaginitis.

I hope that, in addition to drawing needed attention to an important population health issue, this supplement leads clinicians, professional organizations, payers, and policy makers to revisit “business as usual” with an eye toward optimizing health care now and into the future.
